# Attitudes of registered physiotherapists in Israel toward people identifying as lesbian, gay, and bisexual: a cross-sectional survey

**DOI:** 10.1186/s12909-021-03018-7

**Published:** 2021-11-16

**Authors:** Roei Klein, Michal Elboim-Gabyzon

**Affiliations:** Physical Therapy Department, Faculty of Social Welfare and Health Sciences, University of Haifa, 199 Aba Khoushy Ave., Mount Carmel, (Eshkol Bldg., Floor 9, room 910), Haifa, Israel

**Keywords:** LGB, Attitudes, Physiotherapists, Sexual and gender minorities, Health-related quality of life, Curriculum, Heterosexuality

## Abstract

**Background:**

This study aimed to explore the attitudes of registered physiotherapists (PTs) in Israel toward people identifying as lesbian, gay, or bisexual (LGB) and to identify background characteristics associated with their attitudes toward LGB individuals.

**Methods:**

This nationwide study in Israel employed an observational design. Participants completed an anonymous online questionnaire which included demographic characteristics (e.g., age, sex, gender, sexual orientation, religious affiliation, and religiousness) and questions regarding sources of knowledge about LGB individuals, and type of acquaintance with an LGB individual. In addition to two self-assessment questions regarding levels of homophobia (active engagement against LGB individuals) and heterosexism (holding negative opinions regarding LGB individuals) answered on a five point Likert scale (1 – not at all homophobic/ heterosexist, 5- very homophobic/ heterosexist) and the Hebrew version of the Attitudes Toward Homosexuality Scale (ATHS). Participants were recruited through professional organizations, social media, and word of mouth.

**Results:**

The data of 383 registered PTs practicing in Israel were analyzed. The median score regarding level of homophobia and heterosexism was one (“not at all”). ATHS scores ranged between 32 and 110 (out of 110), with a median score of 106 and 41% scoring below the median. Multivariant logistic regression indicated that identifying as men, heterosexual orientation, and religiousness were significantly associated with less positive attitudes toward LGB individuals. Only 2% of the participants reported having been introduced to issues regarding the LGB community during their professional physiotherapy education.

**Conclusions:**

Registered PTs in Israel demonstrated favorable attitudes toward LGB individuals, as reflected both by ATHS scores and levels of self-reported homophobia and heterosexism. Based on the current results regarding sources of knowledge, updating the physiotherapy curriculum to include information regarding sexual orientation and health of the LGB community is suggested.

## Background

Sexual minorities often endure poorer physical, mental, and sexual health compared to heterosexual individuals [1]. People who identify as lesbian, gay, bisexual, transgender, or intersex (LGBTI) experience lifelong institutional and social discrimination in areas such as education, employment, recreational activities, and healthcare facilities and services [2, 3]. Stigmatization and derived negative attitudes toward LGBTI people are prevalent among the general population [4].

There is no consensus in the literature regarding the attitudes and behaviors of healthcare professionals toward sexual minorities. Some studies have reported positive and tolerant attitudes toward LGBTI people among healthcare professionals [5, 6]. However, most studies show that healthcare professionals, such as physicians [7], nurses [7], dentists, mental health professionals [6, 7], athletic trainers [8], and social workers [7] do demonstrate varying degrees of sexual prejudice, negative attitudes, and biased care toward sexual minority patients. The nature of the attitudes may depend on the specific profession of the healthcare provider. For example, psychologists and social workers generally exhibit more positive attitudes toward LGBTI people [7, 9] compared to nurses and physicians [7, 10].

Only a few studies have focused on the attitudes of PTs toward LGBTI individuals [11–13]. Burch [11], who explored the attitudes of various health professionals toward individuals with spinal cord injuries, demonstrated that PTs (*N* = 176) generally exhibited attitudes of tolerance toward patients with diverse sexual orientations. Thus, while 1% of PTs stated that they “find it difficult to have respect” for LGBTI patients, and another 1% reported that they have “full respect,” [11] most participants stated, “I have tolerance” and “some respect” (85 and 13%, respectively). Ross and Setchell focused on the experiences of 108 patients who identify as LGBTI, queer, or related identitieswhile attending physiotherapy [13]. Almost all participants reported at least one of the following situations: erroneous assumptions by the PTs, feelings of discomfort, explicit and implicit discrimination, and a lack of knowledge of their specific health needs [13].

Health care of sexual and gender minorities, therapeutic encounters and cultural competency might be improved by integrating in the curriculum studies of health care professional issues relating the population-specific needs of LGBTI [14]. In a survey exploring the integration of LGBTI-related topics in the curriculum of 229 Director of Physical Therapy programs in United States, it was determined that among 31% of respond, only half reported delivering one or more curricula hours per year on this topic [15]. In addition, the survey results indicate that the barriers to the teaching of LGBTI related topics are lack of time and lack of sufficient faculty training [15] .

Owing to the paucity of available information, we believe it is crucial to explore PTs’ attitudes toward individuals identifying as LGB. Furthermore, it is important to understand the background characteristics of PTs which may relate to their attitudes. Understanding these issues may enhance our ability to promote cultural competence and unbiased care toward sexual minorities, thus promoting optimal physiotherapy care in accordance with accepted bioethical principles [16, 17].

The research questions were:

1. What are the attitudes of registered PTs in Israel toward people identifying as LGB?
2. What background characteristics of PTs relate to their attitudes toward LGB individuals?

## Methods

### Design

This study employed an observational design. An anonymous nationwide online self-report survey using Google Forms was conducted between September 2019 and March 2020. Accompanying online instructions indicated that completing and sending the questionnaire was indicative of informed consent. This study was approved by the Ethics Committee at the Faculty of Social and Health Sciences, at the University of Haifa (number 19/388).

### Participants

Participant recruitment involved the use of social media avenues such as Facebook physiotherapy professional groups, the newsletter of the Israeli Physiotherapy Society, and word of mouth among PTs. All respondents were registered PTs in Israel. There were no restrictions regarding clinical experience and workplace.

### Outcome measures

The following were the outcome measures:

1. A demographic questionnaire regarding sex, gender, age, education, place of residence, sexual orientation, professional experience, religious affiliation, and religiousness was used. It also included questions regarding sources of knowledge of LGB individuals (8 response options), knowing individuals within the LGB community (yes/no response) and types of acquaintance with an LGB individual (total of 7 options including nuclear family, distant social circle, etc.); see Table 2.
2. Two self-assessment questions regarding the individual’s level of homophobia and heterosexism, measured on a five-point Likert scale ranging from one (not at all homophobic/heterosexist) to five (very homophobic/heterosexist). Definitions of the two terms were based on Ben-Ari’s [18] paper and were provided in the survey to ensure that participants understood these terms. Thus, “homophobic” was defined as a person who actively acts against members of the LGB community, and “heterosexist” was defined as a person who holds negative opinions regarding LGB community members but does not actively act against them [18].
3. Attitudes were evaluated using the Hebrew version of the Attitudes Toward Homosexuality Scale (ATHS), originally developed by Kite and Deux [19]. The ATH scale is the only validated and reliable tool in Hebrew focusing on attitudes toward homosexuality. The Hebrew version has previously demonstrated good psychometric properties (internal consistency, Cronbach’s alpha = 0.93, and good test-retest reliability (r = .71)) [20, 21]. These results are compatible with the original version. The Hebrew version of the ATHS includes 22 statements (see Table 3) regarding attitudes toward homosexuality rated on a five-point Likert-type scale ranging from one (“strongly agree”) to five (“strongly disagree”). The overall score ranges from 22 to 110 in the Hebrew version, compared to 21 to 105 in the English version (the Hebrew version includes an additional question (question 22) about acquired immunodeficiency syndrome (AIDS). A higher score on the scale indicates more positive attitudes toward the LGB community. Answers to items 1, 2, 6, 8, 13, 14, 15, 18, 19, 20, and 21 are reverse scored. To the best of our knowledge, there is no cutoff value of the ATHS defining the level of negative/ positive attitudes toward homosexuality.

### Data analysis

Descriptive statistics were calculated for personal characteristics of participants, knowledge source and acquaintance with LBG individuals, heterosexism and homophobia levels, and for the results of the ATHS**.**

Since the distribution of the response score by the participants on the dependent variables (heterosexism levels and for the results of the ATHS) were not uniform along the possible scores, we categorized it for the purpose of further statistical analyses (group comparisons and logistic regression analysis).

The ATHS score, the values were not distributed in an even pattern across the possible ranges of 22 to 110. The majority of participants (over 80%) answered a value higher than 100.

Accordingly, the median score (106) of the ATHS variable was defined as the cutoff point and converted into a categorical score. Scores above or equal to the median response of the sample (≥ 106) were one category, and scores below the median (< 106) were the second category. Comparisons analysis between categorical variables were performed using the Chi-square test and were reported as numbers and percentages (%). This analysis explored the relationships between the categorical outcome measures (sex, gender, educational level, place of residence, and sexual orientation), the ATHS score, and the level of heterosexism.

Results regarding age and years of experience as a PTs were normally distributed. Between-group comparisons of ATHS scores for these variables were performed using the t-test and univariate analysis of variance (ANOVA) for the level of heterosexism. The level of religiousness was non-normally distributed, and group comparisons were performed using the Wilcoxon rank-sum test for the ATHS score and the Kruskal-Wallis test for the level of heterosexism.

Multivariate logistic regression analysis was used for the prediction of positive attitudes toward Homosexuality (total ATHS score ≥ 106) by gender, age, sexual orientation, religiousness, years of experience, educational level, and place of residence. Odds Ratio value above value 1 indicates more positive attitudes.

Significance was established at *p*-values ≤0.05.

## Results

### Participants

The study sample included 383 registered PTs (average age 39.1 ± 9.1 years), with 76% females and 90% identifying as heterosexual. Two participants identified as transgender. Most respondents were Jewish (94%). Overall, 74% identified as secular, 14% as traditional, and 12% as religious. Further details on participants’ personal characteristics are presented in Table 1. Details on source of information regarding the LGB community as well as venues of acquaintance are detailed in Table 2. The majority of respondents (66%) reported that the source of their knowledge was the media (e.g., social media, television, newspapers). Only 2% of the respondents reported having been introduced to this topic during their professional physiotherapy bachelor’s or master’s degrees**.** Most of the PTs reported pervious acquaintance with an individual identifying as LGB (97%). The most common types of acquaintance were through distant social circles (70%) or at work/school/academy (75%).

**Table 1:** Personal characteristics of participants

Characteristics	Participants (***N*** = 383)
**Age,** years, mean ± SD, range	39.1 ± 9.1, 23–69
**Sex**, n (%): Male; Female	91 (24); 292 (76)
**Gender,** n (%): Men; Women	93 (24); 290 (76)
**Sexual orientation, n (%)**	
Heterosexual	346 (90)
Lesbian, gay, and bisexual	37 (10)
**Educational level, n (%)**	
Bachelor’s degree	260 (68)
Master’s degree	111 (29)
Doctorate	12 (3%)
**Religion**	
Judaism	358 (94)
Islam	7 (2)
Christianity	14 (4)
Atheist	3 (1)
Missing	1 (0.3)
**Religiousness**	
Secular	284 (74)
Traditional	54 (14)
Religious	45 (12)
**Work experience,** years**,** mean ± SD, range	12.3 ± 9.4, 0.5–44
**Place of work,** n (%)^a^	
Public outpatient care	163 (37)
Private outpatient care	167 (44)
Acute hospital	54 (14)
Rehabilitation hospital	70 (18)
Child development	74 (19)
Academia	25 (7)
Senior citizens’ homes	18 (5)
Others	29 (8)
**Place of residence n (%)**	
Urban	244 (64)
Other forms of residence (Village, Kibbutz, etc.)	139 (36)

SD: standard deviation

^a^can exceed 100%

**Table 2:** Knowledge source and acquaintance with lesbian, gay, and bisexual individuals of 383 registered physiotherapists, n (%)

Characteristic	Participants number (percentage)
**Sources of knowledge**^**a**^	
Have no knowledge on the topic	8 (2%)
Bachelor’s degree in physiotherapy	5 (1%)
Master’s degree in physiotherapy	5 (1%)
Academic studies, not in physiotherapy	7 (2%)
Non-academic studies	22 (6%)
Personal interest	215 (56%)
Media (e.g., social media, television, newspapers)	254 (66%)
Personal acquaintance	301 (79%)
**Acquaintance with an individual identifying as LGB**	
Yes	372 (97%)
No	11 (3%)
**Type of acquaintance with an LGB individual**^**a**^	
Nuclear family	48 (13%)
Extended family	116 (30%)
Close social circle	178 (47%)
Distant social circle	268 (70%)
Patient(s)	161 (42%)
Work, school, academy	286 (75%)
Other	5 (1%)

LGB: lesbian, gay, and bisexual

^a^can exceed 100%

Research question 1: PTs’ attitudes toward LGB individuals.

Table 3 & Table 4 delineates the results of both the questions regarding levels of homophobia, heterosexism and the ATHS scale.

**Table 3:** Descriptive statistics of heterosexism and homophobia levels (Median, IQR, Distribution per Likert scale; number of respondents (percentage) (N = 383)

Variable	
Total score regarding heterosexism: Median (IQR)	1.0 (1.0,3.0)
Distribution per Likert scale; number of respondents (percentage)	
Level 1(not at all)	239 (62.4%)
Level 2	29(7.57%)
Level 3	32(8.36%)
Level 4	27(7.05%)
Level 5 (very)	56(14.62%)
Total score regarding homophobia: Median (IQR)	1.0 (1.0,1.0)
Distribution per Likert scale; number of respondents (percentage)	
Level 1 (not at all)	342 (89.3%)
Level 2	29 (7.57%)
Level 3	8(2.09%)
Level 4	1(0.26%)
Level 5 (very)	3(0.78%)

**Table 4:** The results of the Attitudes Toward Homosexuality Scale (Median; range; IQR) (N = 383)

Variable	Median; range; (IQR)
**Attitudes Toward Homosexuality Scale**	
Total Attitudes Toward Homosexuality Scale score	106; 32.00–110.00 (102–109)
1. I would not mind having homosexual friends.	5; 1–5 (5–5)
2. Finding out that an artist was gay would have no effect on my appreciation of his/her work.	5; 1–5 (5–5)
3. I would not associate with known homosexuals if I can help it.	5; 1–5 (5–5)
4. I would look for a new place to live if I found out my roommate was gay.	5; 1–5 (5–5)
5. Homosexuality is a mental illness.	5; 1–5 (5–5)
6. I would not be afraid for my child to have a homosexual teacher.	5; 1–5 (5–5)
7. Gays dislike members of the opposite sex.	5; 1–5 (5–5)
8. I do not really find the thought of homosexual acts disgusting.	5; 1–5 (3–5)
9. Homosexuals are more likely to commit deviant sexual acts, such as child molestation, rape, and voyeurism (peeping Toms), than are heterosexuals.	5; 1–5 (5–5)
10. Homosexuals should be kept separate from the rest of society (i.e., separate housing, restricted employment).	5; 1–5 (5–5)
11. Two individuals of the same sex holding hands or displaying affection in public is revolting.	5; 1–5 (5–5)
12. The love between two males or two females is quite different from the love between two persons of the opposite sex.	5.; 1–5 (4–5)
13. I see the gay movement as a positive thing.	5; 1–5 (4–5)
14. Homosexuality, as far as I’m concerned, is not sinful.	5; 1–5 (5–5)
15. I would not mind being employed by a homosexual.	5; 1–5 (5–5)
16. Homosexuals should be forced to have psychological treatment.	5; 1–5 (5–5)
17. The increasing acceptance of homosexuality in our society is aiding in the deterioration of morals.	5; 1–5 (5–5)
18. I would not decline membership in an organization just because it has homosexual members.	5; 1–5 (5–5)
19. I would vote for a homosexual in an election for public office.	5; 1–5 (5–5)
20. If I knew someone were gay, I would still go ahead and form a friendship with that individual.	5; 1–5 (5–5)
21. If I were a parent, I could accept my son or daughter being gay.	5; 1–5 (4–5)
22. Homosexuals are guilty of spreading AIDS.	5; 1–5 (4–5)

Notes: We used the validated Hebrew version but are presenting here the original English questions; questions 1, 2, 6, 8, 13, 14, 15, 18, 19, 20, and 21 were reverse scored. Question 22 was added and validated for the Hebrew version of the questionnaire

IQR: interquartile range, SD: standard deviation; AIDS: acquired immunodeficiency syndrome

The median level of homophobia score was one out of five, indicating extremely low levels of homophobia. The distribution of the answers in the five-point Likert was unequal across the scale, with 89% scoring one (not at all homophobic) and 8% scoring two. Accordingly, this outcome measure was not included in further statistical analyses (group comparisons and logistic regression analysis).

The median level of heterosexism score was one out of five, indicating extremely low levels of heterosexism. The distribution of the answers regarding heterosexism level were more balanced compared to the level of homophobia with 62.4% indicating a score of 1, and remaining distributed along the scale (for details see Table 3). Thus, the level of heterosexism was categorized as low (scores 1–2), medium (score 3), and high (scores 4–5).

The median ATHS score was 106 out of 110 indicating favorable attitudes toward LGB individuals. Scores ranged between 32 and 110, with 41% scoring below the median.

Research question 2: Relation of background characteristics with PTs’ attitudes.

The results of the relation of different background characteristics with levels of heterosexism as well as the ATHS scores are presented in Table 5.

**Table 5:** Comparative analysis of background characteristics and attitudes toward homosexuality

		ATHS score			Levels of heterosexism			
		< 106 (***n*** = 158)	≥ 106 (***n*** = 225)	***P*** value	1/2 (***n*** = 268)	3 (***n*** = 32)	4/5 (***n*** = 83)	p value
Sex, n (%)^a^	Male	53 (34)	38 (17)	< 0.01	53 (20)	11 (34)	27 (32)	0.02
	Female	105 (66)	187 (83)		215 (80)	21 (66)	56 (68)	
Gender, n (%)^a^	Men	54 (34)	39 (17)	< 0.01	55 (21)	11 (34)	27 (32)	0.03
	Women	104 (66)	186 (83)		213 (79)	21 (66)	56 (68)	
Education MA/PhD, n (%)^a^	Yes	52 (33)	71 (32)	0.78	87 (33)	10 (31)	26 (31)	0.98
	No	106 (67)	154 (68)		181 (67)	22 (69)	57 (69)	
Place of residence, n (%)^a^	Urban	100 (63)	144 (64)	0.89	176 (66)	17 (53)	51 (62)	0.34
		58 (37)	81 (36)		92 (34)	15 (47)	32 (38)	
Sexual orientation, n (%)^a^	Not LGB	151 (96)	195 (87)	< 0.01	235 (88)	29 (91)	82 (99)	0.01
	LGB	7 (4)	30 (13)		33 (12)	3 (9)	1 (1)	
Religiousness, n (%)^b^	Secular	89 (56)	195 (87)	< 0.01	216 (80)	18 (56)	50 (60)	< 0.01
	Traditional	32 (20)	22 (10)		31 (12)	6 (19)	17 (21)	
	Religious	37 (24)	8 (3)		21 (8)	8 (25)	16 (19)	
Age (years), mean ± SD^c^		38.1 ± 9.1	39.8 ± 9.0	0.06	39.0 ± 9.0	38.2 ± 9.3	39.8 ± 9.3	0.6
Years of experience, mean ± SD^c^		11.4 ± 9.2	12.8 ± 9.4	0.16	12.1 ± 9.2	11.91 ± 9.6	12.9 ± 9.8	0.6

ATHS: Attitudes Toward Homosexuality Scale; SD: standard deviation; LGB: lesbian, gay, and bisexual

^a^Chi-square test, ^b^Wilcoxon and Kruskal-Wallis tests, ^c^t-test and univariate ANOVA

Males and men demonstrated significantly less favorable attitudes toward homosexuality, as reflected by the higher levels of heterosexism and the number/percentage of participants scoring below the median on the ATHS, as compared to females and women (17% males versus 83% females for > 106 on the ATHS) Conversely, age, educational level, years of experience, and place of residence made no difference in the participants’ attitudes toward LGB individuals based on analyses for heterosexism level question or the ATHS scores. Participants belonging to sexual and gender minority groups had more positive attitudes toward LGB people (87% not LGB versus 13% LGB for > 106 on the ATHS). Individuals identifying as secular demonstrated more positive attitudes than traditional and religious individuals (87% secular versus 13% traditional and religious for > 106 on the ATHS).

Logistic regression results indicated that gender, sexual orientation, and religiousness significantly predicted attitudes toward LGB individuals, as reflected in the ATHS scores; for details, see Table 6. Woman, non-heterosexual orientation, and secularity and being traditional were predictors of positive attitudes toward LGB individuals (ATHS value ≥106). The probability of women achieving ATHS value ≥106 was 4.11 times greater than the probability of men (OR 4.11, 95% CI 2.32 to 7.27). For persons of non-heterosexual orientation, the chance of achieving ATHS value ≥106 is 4.58 times greater than those of heterosexual orientation (OR 4.58, 95% CI 1.76 to 11.89). A secular person’s chances of achieving ATHS value ≥106 was 10.70 times greater than a religious person’s (OR 10.7, 95% CI 4.55 to 25.17). A traditional person’s chance of achieving ATHS value ≥106 was 2.92 times greater than a religious person’s (OR 2.92, 95% CI 1.10 to 7.74).

**Table 6:** Multivariance logistic regression analysis results for the prediction of Attitudes Toward Homosexuality Scale scores ≥106, indicating a positive attitude (odds ratio, confidence interval)

	ATHS Score (≥ 106)
Age	1.08 (0.98–1.19)
Gender (woman)	4.11 (2.32–7.27) ***
Sexual orientation (not heterosexual)	4.58 (1.76–11.89) **
Religiousness (secular)	10.70(4.55–25.17) ***
Religiousness (traditional	2.92 (1.10–7.74) **
Years of experience	0.93 (0.85–1.03)
Educational level (not holding MA/PhD)	1.23 (0.72–2.07)
Place of residence (not urban)	1.39 (0.84–2.29)
Wald (df)	61.3 (8) ***
C statistic	0.76

*p value < 0.05, **p value < 0.01, ***p value < 0.0001

These three variables accounted for 61% of the variance (see Table 6).

## Discussion

This is the first study that explores the attitudes of registered PTs in Israel toward people identifying as LGB. PTs in Israel demonstrated considerably positive attitudes toward LGB individuals, as reflected by their high ATHS scores. Few studies have focused on examining PTs’ attitudes toward the LGBTI community [11–13]. Since none of these studies used the ATHS questionnaire, it is not possible to compare the results of the present study to them.

LGBTI individuals have reported inappropriate experiences in terms of their personal comfort, discriminative behavior, and professional understanding of their health needs during physiotherapy treatment [13]. Burch’s study focusing on the perspective of PTs indicates tolerant attitudes toward LGB patients of spinal cord injuries [11]. Copti et.al. (2016) [12] recommended, in a position paper, to ensure training of sexual orientation diversity competency during the physiotherapy education programs including self-awareness of implicit and explicit bias toward the LGBTI community and the possible effect on their attitude and mode of treatment, appropriate usage of language, and learning the specific health needs. Similarly, Glick et.al (2020) [15] focused on the physiotherapy education curricula in the US regarding LGBTI health topics and recommended updating the existing curricula, and increasing the competency of the physiotherapy students to prevent discriminatory care.

ATHS scores in the present study were higher than those previously reported by Shilo [20], who targeted social work students in Israel. However, it is unlikely that this difference between studies is indicative of true differences in the attitudes of PTs versus social workers. In fact, previous studies consistently indicated more positive attitudes toward sexual minorities among social workers in comparison to other health professionals [7, 9, 22]. In general, changes such as secularization and the increased demand for individual equality might have increased acceptance of sexual diversity in various areas of life [23]; thus, the more positive attitudes reported in this study may reflect the progressive social, cultural, and legislative changes toward sexual minorities that have occurred in Israel since the publication of Shilo’s study in 2004 [20].

The majority (97%) of the participants reported low or very low levels of homophobia, thus indicating that they would not engage in overt activities against members of the LGB community. In contrast, 22 % of the participants stated that they held negative opinions regarding LGB individuals, as reflected by high or very high levels of heterosexism. This disparity may be explained by considering the concepts of homophobia and heterosexism as two ends of a continuum representing negative attitudes and behaviors toward the LGB community [24]. While the high ATHS scores and low self-reported levels of homophobia may reflect participants’ explicit attitudes, their responses to the question regarding heterosexism may be more representative of implicit attitudes, which are far less positive. Such a disparity between implicit and explicit attitudes toward the LGB community has been reported in previous studies, as well [25]. The survey was kept anonymous to encourage truthful and honest responses. Anonymity was particularly important to increase the responsiveness of PTs belonging to more traditional and religious sections of the population, where issues relating to the LGB community are still considered taboo. However, as the questionnaires were distributed through professional and formal channels, this may have led to participants feeling obliged to comply with societal expectations and the ethical codes of the physiotherapy profession, which stress a commitment to providing unbiased healthcare to all minority groups [17]. In addition, it is possible that the explicit nature of most questions led the responder to tone down their attitudes due to self-awareness and fear that negative attitudes towards the LGB community may be perceived by the researcher as backward. However, it is also likely that some of the more traditional PTs simply ignored the questionnaire as they may have considered it an issue that should remain taboo.

The current multivariant logistic regression analysis demonstrated that gender, sexual orientation, and religiousness are significantly predictable with attitudes toward LGB individuals, as reflected in the ATHS scores. Those results are consistent with previous studies [7, 26–29]. Gender has previously been shown to be a significant predictor of attitudes toward LGB individuals, with men exhibiting more homophobic attitudes [26]. Religion is another well-established predictor of negative attitudes toward LGB people, sexual prejudice, and acts of discrimination against the LGB community [26, 27]. Thus, religious healthcare professionals have been known to exhibit more negative attitudes toward LGB individuals [26, 28]. Previous studies also support the association between the sexual orientation of healthcare providers and their attitudes toward LGB individuals, with healthcare professionals who identify as heterosexuals reporting more negative implicit attitudes compared to sexual minority healthcare providers [7, 29].

The registered PTs participants in the current study were also asked about their source of knowledge about the LGB community. The findings of the study were that only 2% of the participants stated that they had no knowledge regarding the LGB community. Although this finding seems very promising, the source of this information was not professional based on the current results. Only 2% of the respondents reported having been introduced to this topic during their professional physiotherapy bachelor’s or master’s degrees. Furthermore, the majority of respondents (66%) reported that the source of their knowledge was the media (e.g., social media, television, newspapers). The present study did not examine the relation between the PTs attitudes toward LGB individuals and their sources of knowledge regarding LGB individuals and types of acquaintance with an LGB individual. Further studies should address this point. Knowledge regarding sexual orientation has been demonstrated to be a crucial factor influencing professional healthcare providers’ attitudes toward people identifying as LGB [30]. Previous studies indicate that the knowledge deficits of healthcare professionals may lead to failure to inquire about their patients’ sexual orientation, gender identity, and sexual health [31]. Inequalities and disparities in the physical and mental health status of sexual and gender minorities and in health care as experienced by sexual and gender minorities are well documented [32]. This undesirable situation a product of knowledge deficit of healthcare professionals, misunderstanding and non-recognition of the unique needs of LGBTI individuals, institutional and personal discrimination and phobia, and stigmatization and negative attitude of health care professional [33]. These current results highlight the need to address the issue of knowledge deficit of healthcare professionals by updating the physiotherapy curriculum and designing postgraduate education courses that include knowledge regarding sexual orientation and its effect on mental and physical health issues as well as the specific needs of the LGB community.

Several study limitations must be considered. The questionnaires distributed in this study addressed only PTs’ attitudes toward LGB individuals and not their attitudes toward a wider range of sexual and gender minorities. The attitudes presented here relate only to registered PTs in Israel. Therefore, the results may not be representative of PTs belonging to other countries and cultures. Finally, compliance percentages could not be calculated as the questionnaires were distributed online and the total number of PTs who chose not to complete the questionnaires could not be obtained Thus, our recruitment method (via social media and snowballing method) may have implications on the representativeness of the population of interest, increasing the possibility that those who felt particularly strongly about the topic were our primary respondents.

## Conclusions

Registered PTs in Israel demonstrate favorable attitudes toward LGB individuals. PTs who identify as men, heterosexual, and religious or traditional are more likely to hold negative attitudes toward LGB individuals. Only 2% of the participants reported physiotherapy studies as a source of knowledge on sexual and gender minorities. Despite the relatively positive attitudes of PTs towards sexual minorities, reliable knowledge regarding the special health needs of sexual minorities should be addressed both during physiotherapy educational programs and in post graduate professional courses. Further studies are required to explore how implicit and explicit attitudes toward people identifying as LGB affect physiotherapy treatment quality.

## Data Availability

The data are available from the corresponding author on reasonable request.
